# A Case of Vesico-Vaginal Fistula Remedied by Caustic

**Published:** 1847-10

**Authors:** Elan W. Harris

**Affiliations:** Elm Wood, Cape Girardeau county, Mo.


*A Case of Vesico-Vaginal Fistula remedied by Caustic.* By Elan W. Harris, M. D., of Elm Wood, Cape Girardeau county, Mo.—Mrs. C., a married lady, aged 30 years, presented herself to me early in February last, laboring under the unfortunate, painful, and disgusting infirmity of vesico-vaginal fistula. Her garments were constantly wet; the vaginal cavity, labia and thighs bathed with urine, in an erysipelatous condition, and exquisitely tender. The complaint had existed for five years, and occurred seven or eight days after a tedious first labor, and violently severe manipulations of her midwife. In addition to the soreness caused by the irritation of the urine, she suffered violent pain in the bladder, which often prevented sleep whole nights; she sometimes passed urine the natural way for a day or two at a time, but always with great pain, and if, during her monthly periods, the urine is discharged through the urethra it is mixed with the catamenial fluid, just as it is when the urine passes through the fistula and vagina.

The parts being too sore to attempt any exploration, recumbency, aperients, fomentation, and tepid lotions were enjoined. In a few days, her condition being much improved, the finger was introduced into the vagina, the walls of which felt hard and irregular, presenting to the finger the sensation of cicatrices. No os tincæ or neck of the womb could be felt. The vaginal speculum was now carefully inserted into the vagina, which terminated in a round sack-like cavity without anything like the neck of a womb projecting into it. Instead of an os tincæ, a small opening was found large enough to admit a silver probe which entered the uterus; about three quarters of an inch from this aperture, in the anterior wall of the vagina, was found an oblique fistulous opening into the bladder (five lines in extent), through which the urine could be seen flowing. The bladder was then sounded, and I soon convinced myself of the existence of a calculus. The patient was informed that the only relief that could be afforded was by extracting the stone, and that there was barely a hope that the fistula might be healed, and thereby relief obtained from the troublesome and disgusting incontinence. She replied that she would willingly submit to *any* operation rather than remain in her miserable condition.

On the second day after the examination, a long delicate pair of forceps was introduced through the meatus urinarius, with a bistoury at hand to make the proper incision if found to be necessary for the extraction of the stone. By gently and gradually opening the chaps of the forceps, the urethra was sufficiently dilated in about twelve minutes, (with very little pain), to enable me to take hold of the stone. In endeavoring to get a firm grasp, this was crushed to pieces, which I considered a fortunate occurrence; the fragments were removed with the forceps and syringe, at a sitting of a few minutes each day for five days, when no more could be found. Some of the particles passed through the fistula and were washed out of the vagina with a syringe. I weighed four drachms and six grains of gravel saved, and there was fully as much lost. No unpleasant symptom occurred, and she was permitted to walk about, expressing great gratification on account of freedom from pain.

The incontinence was still annoying, and on the sixth day after the removal of the last of the gravel, the speculum was again introduced, and a piece of solid lunar caustic made fast by a thread in the same forceps used for extracting the stone, was carried up through the speculum into the vesico-vaginal opening, and rubbed on the edges and angle of the wound, until they were completely cauterized. A tube was firmly fixed in the bladder to conduct the urine into a vessel placed below, and emollient injections daily used. On the third day there was tenderness and pain in the pelvic region, fever and bilious vomiting. The catheter was removed, and by the use of the lancet, an emetic, and fomentations, those symptoms were relieved. The catheter was then replaced and not removed until the eighth day, when to my great satisfaction it was found that she could retain the urine and pass it per vias naturales.

Thus has a cure been obtained of a loathsome malady, which at a time still not very remote was supposed to be beyond the resources of art.

I saw Mrs. C., yesterday, and she informed me that she had menstruated three times since the operation without difficulty or pain, but that the menstrual fluid passes from the bladder mingled with the urine, showing, I think, the existence of utero-vesical fistula. Fears are entertained that particles of the menstrual fluid retained in the bladder may form the nucleus of another stone. The woman, however, is contented and happy.—*Western Journal of Medicine and Surgery.*

